# Effects of the (Pro)renin Receptor on Cardiac Remodeling and Function in a Rat Alcoholic Cardiomyopathy Model via the PRR-ERK1/2-NOX4 Pathway

**DOI:** 10.1155/2019/4546975

**Published:** 2019-03-13

**Authors:** Xinran Cao, Shiran Yu, Yuanyuan Wang, Min Yang, Jie Xiong, Haitao Yuan, Bo Dong

**Affiliations:** ^1^Department of Cardiology, Shandong Provincial Hospital Affiliated to Shandong University, Jinan 250012, China; ^2^The Key Laboratory of Cardiovascular Remodeling and Function Research, Chinese Ministry of Education, Chinese National Health Commission and Chinese Academy of Medical Sciences, The State and Shandong Province Joint Key Laboratory of Translational Cardiovascular Medicine, Department of Cardiology, Qilu Hospital of Shandong University, Jinan 250012, China; ^3^The Third Hospital of Jinan, Jinan 250012, China

## Abstract

Alcoholic cardiomyopathy (ACM) caused by alcohol consumption manifests mainly as by maladaptive myocardial function, which eventually leads to heart failure and causes serious public health problems. The (pro)renin receptor (PRR) is an important member of the local tissue renin-angiotensin system and plays a vital role in many cardiovascular diseases. However, the mechanism responsible for the effects of PRR on ACM remains unclear. The purpose of this study was to determine the role of PRR in myocardial fibrosis and the deterioration of cardiac function in alcoholic cardiomyopathy. Wistar rats were fed a liquid diet containing 9% *v*/*v* alcohol to establish an alcoholic cardiomyopathy model. Eight weeks later, rats were injected with 1 × 10^9^v.g./100 *μ*l of recombinant adenovirus containing EGFP (scramble-shRNA), PRR, and PRR-shRNA via the tail vein. Cardiac function was assessed by echocardiography. Cardiac histopathology was measured by Masson's trichrome staining, immunohistochemical staining, and dihydroethidium staining. In addition, cardiac fibroblasts (CFs) were cultured to evaluate the effects of alcohol stimulation on the production of the extracellular matrix and their underlying mechanisms. Our results indicated that overexpression of PRR in rats with alcoholic cardiomyopathy exacerbates myocardial oxidative stress and myocardial fibrosis. Silencing of PRR expression with short hairpin RNA (shRNA) technology reversed the myocardial damage mediated by PRR. Additionally, PRR activated phosphorylation of ERK1/2 and increased NOX4-derived reactive oxygen species and collagen expression in CFs with alcohol stimulation. Administration of the ERK kinase inhibitor (PD98059) significantly reduced NOX4 protein expression and collagen production, which indicated that PRR increases collagen production primarily through the PRR-ERK1/2-NOX4 pathway in CFs. In conclusion, our study demonstrated that PRR induces myocardial fibrosis and deteriorates cardiac function through ROS from the PRR-ERK1/2-NOX4 pathway during ACM development.

## 1. Introduction

Alcohol consumption can lead to serious cardiovascular complications, particularly alcoholic cardiomyopathy (ACM), which is a specific heart muscle disease. ACM is characterized mainly by interstitial fibrosis and maladaptive myocardial function that eventually evolves into heart failure [1]. Among male patients with alcohol abuse, alcohol intake is considered to be one of the most important causes of nonischemic dilated cardiomyopathy [2]. Although the exact mechanism of ACM remains unclear, it has been widely accepted that oxidative stress plays an important part in the development of ACM [3]. Jing et al. [4] reported that alcohol intake increased the oxidative stress level in alcoholic hearts accompanied by cardiomyocyte apoptosis. Tan et al. [5] also found that alcohol induced cardiac nitrative damage and remodeling in a protein kinase C/oxidative stress manner. These studies have noted that oxidative stress caused by alcohol intake increases myocardial fibrosis and exacerbates cardiac function impairment.

There is ample evidence to prove that the renin-angiotensin system (RAS), as one of the most important modulators in ROS production, plays a crucial role in the development of ACM [5]. The (pro)renin receptor (PRR) is an important member of local tissue RAS, and the biological function has been controversial since its first discovery by Nguyen et al. in 2002 [6]. PRR can bind to both (pro)renin and renin, which activates the classical renin-angiotensin pathway by increasing the production of angiotensin II (Ang II). On the other hand, this binding process activates the receptor itself, which also activates other intracellular signaling pathways that are independent of Ang II [7, 8], including increased profibrotic and proinflammatory factors production and cell proliferation [9]. Lian et al. found that heart-specific overexpression of PRR induced atrial fibrillation in mice accompanied by the activation of ERK1/2 [10]. Mahmud et al. reported that the expression level of PRR significantly increased in animal models of myocardial infarction. Moreover, an increase in PRR expression was shown in endomyocardial biopsy samples from patients with dilated cardiomyopathy. [11]. Therefore, it seems that PRR knockout genetic animal models are particularly important in the study of PRR function. However, cardiomyocyte-specific knockout of PRR resulted in mice dying within 3 weeks because of lethal heart failure [12]. In addition, smooth muscle cell-specific knockout of PRR results in nonatherogenic sclerosis due to the dysfunction of V-ATPase and cell recycling in the abdominal aorta [13].

The gene encoding PRR is named *ATP6ap2*, because PRR was originally discovered as a V-ATPase membrane-associated protein [14]. Subsequent studies have shown that PRR does not modulate V-ATPase activity, but is essential for the assembly of V-ATPase subunits [15]. V-ATPase is involved in many important cellular processes such as acidification, transport, fusion events, and autophagy [9, 16]. With regard to autophagy, dysfunction of V-ATPase activity induced by PRR deletion leads to aggregation of autophagosomes and a blockade of autophagy flux [12]. In addition, PRR can function as an adaptor protein that links V-ATPase and low-density lipoprotein receptor-related protein 6 (LRP6), a coreceptor of Wnt receptor Frizzled. In this way, PRR is involved in signal transduction of the Wnt signaling pathway. Liu et al. reported that PRR aggravated kidney damage and the progression of fibrosis as an amplifier of the Wnt signaling pathway [17, 18].

A number of researchers have studied the relationship between PRR and diseases owing to its important biological functions. However, few researchers have studied the relationship between PRR and ACM. In the present study, we hypothesized that PRR may increase myocardial fibrosis and impair cardiac function through ERK1/2-NOX4-derived reactive oxygen species. Therefore, a series of in vivo and in vitro experiments were conducted to clarify this hypothesis.

## 2. Materials and Methods

### 2.1. Recombinant Adenovirus Generation

The rat PRR cDNA sequence was synthesized as described in GenBank (gene ID: 302526). The product was cloned into pDC315 with the *EcoR* I and *Sal* I sites. Recombinant adenovirus (Ad) carrying the rat PRR or a control transgene was generated as described using the AdMax system [19].

### 2.2. PRR Silencing by PPR-shRNA

Recombinant adenovirus vectors that expressed PRR shRNA were designed by GenePharma (Shanghai GenePharma Co. Ltd, Shanghai, China). Three different interfering sequences targeting PRR were synthesized as follows:

Number 1: 5′-GCTTGCTGTGGGCAACCTATT-3′

Number 2: 5′-GCTCCGTAATCGCCTGTTTCA-3′

Number 3 : 5′-GCATCTCGCCAAGGATCATTC-3′

RT-PCR and western blot were used to select the most efficient interference sequence. Scramble-shRNA served as the negative control.

### 2.3. Animal Model

A total of 90 male Wistar rats (Shandong University Animal Center, China) were randomly divided into the control group (fed with normal diet, *n* = 15) and the model group (fed with alcohol liquid diet, *n* = 75). The alcohol liquid diet contained 9% *v*/*v* alcohol. 8 weeks later, the blood alcohol concentration reached 237 ± 42 mg, which can be considered alcoholic status [20]. The rats whose echocardiographic values tended to be greater and cardiac systolic function decreased were considered as the alcoholic cardiomyopathy model. The rats suffering alcoholic cardiomyopathy were further divided into (i) ACM group (*n* = 15), (ii) EGFP group (*n* = 15), (iii) Scramble-shRNA group (*n* = 15), (iv) PRR group (*n* = 15), and (v) PRR-shRNA group (*n* = 15). Rats in the indicated groups were injected with 1 × 109v.g./100 *μ*l (dissolved in normal saline) of recombinant adenovirus containing EGFP, PRR, Scramble-shRNA, and PRR-shRNA via the tail vein.

4 weeks after the recombinant adenovirus injection, all rats were euthanized and sacrificed. The myocardium of the left ventricle was collected for further experiments. All animal protocols were conducted in accordance with National Institutes of Health Guidelines on the Care and Use of Laboratory Animals. All procedures were approved by the Animal Care and Use Committee of Shandong Provincial Hospital, Shandong University.

### 2.4. Echocardiography

At the end of week 8 and week 12, the rats in all of the groups were anesthetized by intraperitoneal injection of pentobarbital (60 mg/kg). The hemodynamic parameters of the rats in all groups (control group, ACM group, EGFP group, Scramble-shRNA group, PRR group, and PRR-shRNA group) were measured by transthoracic echocardiographic scanning using the Vevo 770 imaging system equipped with a 25 MHz transducer (Visual Sonics, Toronto, Canada). Left ventricular function including left ventricular ejection fraction (LVEF), fractional shortening (FS), left ventricular end-diastolic diameter (LVEDD), left ventricular end-systolic diameter (LVESD), and the ratio of the early peak to the late peak (E/A ratio) was measured. An average of 3 consecutive cardiac cycles was performed on all measurements by an experienced technician blinded to the rat group.

### 2.5. Histopathology and Immunohistochemistry

The myocardium of the left ventricle was fixed with 4% paraformaldehyde, dehydrated with an ethanol gradient and embedded in paraffin. 4.5 *μ*m-thick tissue sections were prepared. Hematoxylin and eosin (HE) staining and Masson's trichrome staining were performed to evaluate the cardiomyocyte cross-section area and myocardial interstitial fibrosis. Immunohistochemical staining was performed as previously described [21]. Briefly, serial sections were deparaffinized and rehydrated, then incubated with anti-rat collagen I and III (both 1 : 250, Abcam, UK), CTGF (1 : 100, Boster, China), and NOX4 (1 : 200, Abcam, UK). Secondary antibody incubation and DAB color development were performed as in the manufacturer's instructions (Zsgb Bio, China). All histopathological sections were photographed and analyzed by a customized imaging analysis system (Image-Pro Plus_6.0, Media Cybernetics, Rockville, MD, USA).

### 2.6. Cell Culture

In order to evaluate the in vitro effects of PRR overexpression on alcohol-induced injury in neonatal cardiac fibroblasts (CFs), cells were seeded in 6-well plates and transfected with Ad-PRR or Ad-EGFP (multiplicity of infection = 150). We administrated angiotensin II type 1 (AT1) receptor antagonist telmisartan (Tel, MCE, Monmouth Junction, USA, 10 *μ*M) and (or) PD98059 (Selleck, Houston, USA, 20 *μ*M) to inhibit ERK kinase 30 min before alcohol stimulation. The CFs were randomly divided into 7 groups and exposed as follows: control group (without alcohol), alcohol group (with alcohol, 200 mM), alcohol+Ad-EGFP (EGFP) group, alcohol+Ad-PRR (PRR) group, alcohol+Ad–PRR+Tel (PRR+Tel) group, alcohol+Ad–PRR+PD98059 (PRR+PD98059) group, and the alcohol+Ad–PRR+Tel+PD98059 group (PRR+Tel+PD 98059). The cells and culture medium were collected for further experiment after 24 h.

In order to evaluate the in vitro effects of PRR-shRNA-mediated PRR silencing on alcohol-induced injury of neonatal cardiac fibroblasts (CFs), cells were seeded in 6-well plates and transfected with Ad-PRR-shRNA or Ad-Scramble-shRNA (multiplicity of infection = 150). The CFs were randomly divided into 4 groups: control group (without alcohol), alcohol group (with alcohol, 200 mM), alcohol+Ad-Scramble-shRNA (Scramble-shRNA) group, and the alcohol+Ad-PRR-shRNA (PRR-shRNA) group. The cells and culture medium were collected for further experiments after 24 h.

### 2.7. Dihydroethidium Fluorescence and NADPH Oxidase Activity

The oxidative stress levels of myocardial tissue and primary rat neonatal cardiac fibroblasts in vitro were measured. Increased production of reactive oxygen species (ROS) was measured using fluorescent dye dihydroethidium (DHE). Briefly, fresh frozen myocardial sections were washed with phosphate-buffered saline (PBS) and incubated with DHE (10 *μ*M) at 37°C for 30 min. For cultured CFs, cells were washed with Dulbecco's modified Eagle's medium (DMEM, Gibco BRL, Gaithersburg, MD) and incubated with DHE (10 *μ*M) at 37°C for 30 min. The fluorescent images of tissue sections and cells were observed (excitation 520 nm and emission 590 nm) and analyzed by fluorescence microscope (Olympus, Japan). The activity of NADPH oxidase in CFs was also determined using an assay kit (Nanjing Jiancheng Bioengineering Institute, China) following the manufacturer's instructions.

### 2.8. Western Blot Analysis

Total proteins from the myocardium and CFs were extracted. Briefly, the myocardium and CFs were lysed in RIPA buffer containing a protease inhibitor cocktail. The lysate was centrifuged at 12000 rpm for 15 minutes at 4°C. Supernatants were incubated with loading buffer and boiled for 5 min. Proteins were separated by SDS-PAGE gel and transferred to polyvinylidene difluoride membranes. The membranes were blocked with 5% skim milk before incubation with specific antibodies. The membranes were incubated with anti-rat PRR, NOX4, TGF-*β* (Abcam, Cambridge, UK), and anti-rat ERK and p-ERK (Cell Signaling Technology, Boston, USA) using the recommended concentration overnight at 4°C. The membranes were incubated with horseradish peroxidase-conjugated secondary antibodies for 2 hours at room temperature. The intensity of bands was quantitated by ImageJ (National Institutes of Health, Bethesda, MD, USA). Protein levels were normalized to GAPDH.

### 2.9. ELISA

Soluble collagen I and collagen III (collagen I and collagen III rat ELISA kits, USCN Life Sciences, Wuhan, China) and transforming growth factor-*β* (TGF-*β*) (TGF-*β*1 rat ELISA kit, Abcam, Cambridge, UK) in the media of the cardiac fibroblasts were measured as we described previously [22].

### 2.10. Real-Time PCR

Total RNA was extracted from CFs by TRIzol (Invitrogen, USA). cDNA was synthesized using SuperScriptIII (Takara, Japan). cDNA was amplified by use of SYBR Green (Takara, Japan). Relative gene expression levels were quantitatively assessed using the 2^-△△CT^ method. Primers for PRR and the housekeeping gene GAPDH were as follows: rat PRR forward 5′-TCTGTTCTCAACTCGCTCCC-3 and reverse 5′-TCTCCATAACGCTTCCCAAG-3′ and rat GAPDH forward 5′-TCTCTGCTCCTC CCT GTTCT-3′ and reverse 5′-ATCCGTTCACACCGACCTTC-3′.

### 2.11. Statistical Analysis

All data were expressed as means ± SE and evaluated by the use of SPSS 17.0 (SPSS Inc., Chicago, IL, USA). Intergroup data were compared by one-way ANOVA. *P* < 0.05 was considered statistically significant.

## 3. Results

### 3.1. PRR Overexpression Induced Myocardial Hypertrophy and Increased Phosphorylation of ERK 1/2 and Expression of NOX4

**Figure 1:**
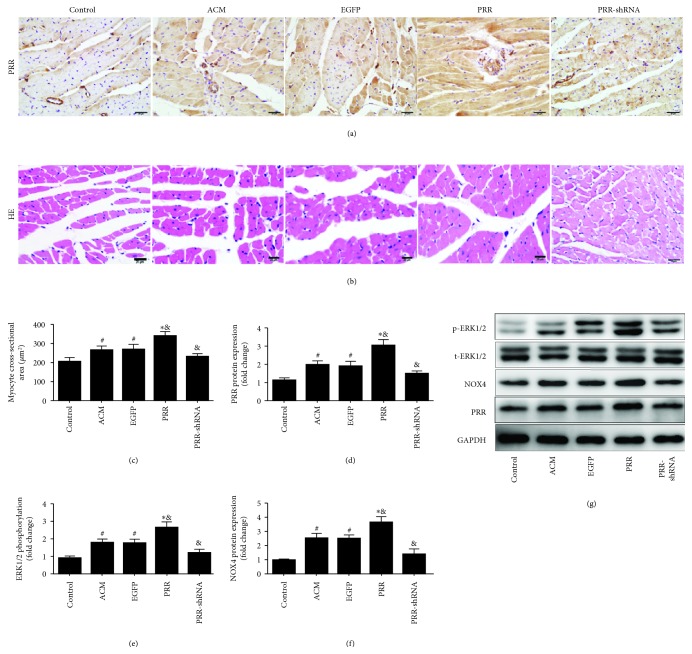
Levels of protein expression and cardiac hypertrophy in all rat groups. (a) Quantitative analysis of PRR protein expression by immunohistochemical staining (scale bar = 20 *μ*m). (b) Representative hematoxylin and eosin staining of cardiomyocyte. (c) Quantitative analysis of cardiomyocytes cross-sectional area (scale bar = 20 *μ*m). (d)–(g) Western blot and statistical analysis of protein expression of GAPDH, PRR, NOX4, and ERK1/2. ^∗^ *P* < 0.01 and ^#^ *P* < 0.05 versus the control group, ^&^ *P* < 0.01 versus the ACM group.

We examined the expression of the (pro)renin receptor (PRR) in all of the rat groups. Compared with the ACM and EGFP groups, western blot and immunohistochemistry showed that PRR expression in the PRR group was significantly increased after 4 weeks of PRR transfection (Figures 1(a) and 1(g)). Our results also revealed an increased expression of PRR in the ACM and EGFP groups than in the control group (Figures 1(a) and 1(g)). Meanwhile, HE staining showed that PRR overexpression prominently increased the cardiomyocyte cross-sectional area in the PRR group, compared with the ACM and EGFP groups (Figure 1(b)). However, there was no significant difference between the ACM group and the EGFP group. Likewise, western blot results showed that PRR overexpression increased ERK 1/2 phosphorylation and protein expression levels of NOX4 in myocardium homogenates (Figure 1(g)). However, these effects were reversed in rats of the PRR-shRNA group compared with the ACM group.

### 3.2. PRR Overexpression Deteriorated Cardiac Function

**Figure 2:**
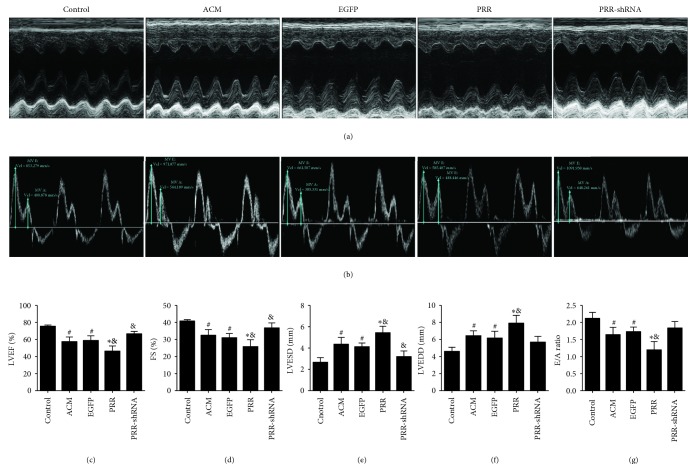
Cardiac function was evaluated by echocardiograms in all rats groups. (a) Representative M-mode echocardiograms. (b) Representative pulsed-wave Doppler echocardiograms of mitral inflow. (c)–(g) Statistical results: LVEF, FS, LVEDD, LVESD, and E/A ratio. LVEF: left ventricle ejection fraction; FS: fractional shortening; LVEDD: left ventricular end diastolic diameter; LVESD: left ventricular end systolic diameter; E/A ratio: early to late mitral flow ratio. .^∗^ *P* < 0.01 and ^#^ *P* < 0.05 versus the control group. ^&^ *P* < 0.05 versus the EGFP group.

At the end of the experiment, transthoracic echocardiography was performed to evaluate cardiac function. Our results revealed that alcoholic rats showed cardiac dilatation accompanied by cardiac dysfunction. Left ventricular end-systolic diameters (LVESD) and left ventricular end-diastolic diameters (LVEDD) were markedly increased. However, left ventricular ejection fraction (LVEF) and fractional shortening (FS) were decreased, as displayed by the M-mode echocardiograms. Accompanying the decrease of systolic function, cardiac diastolic function degraded as indicated by a decrease in the E/A ratio (Figures 2(a)–2(g)). Compared with the control group, all echocardiographic values of the ACM group reflected a decline in cardiac function. Meanwhile, PRR overexpression further aggravated the cardiac dysfunction in the PRR group in contrast to those in ACM and EGFP groups (Figures 2(a)–2(g)). Although PRR silencing decreased LVEDD values and increased E/A values, our results did not show statistically significant differences between the ACM and PRR-shRNA groups (Figures 2(a)–2(g)).

### 3.3. PRR Overexpression Increased Myocardial Fibrosis and Oxidative Stress

**Figure 3:**
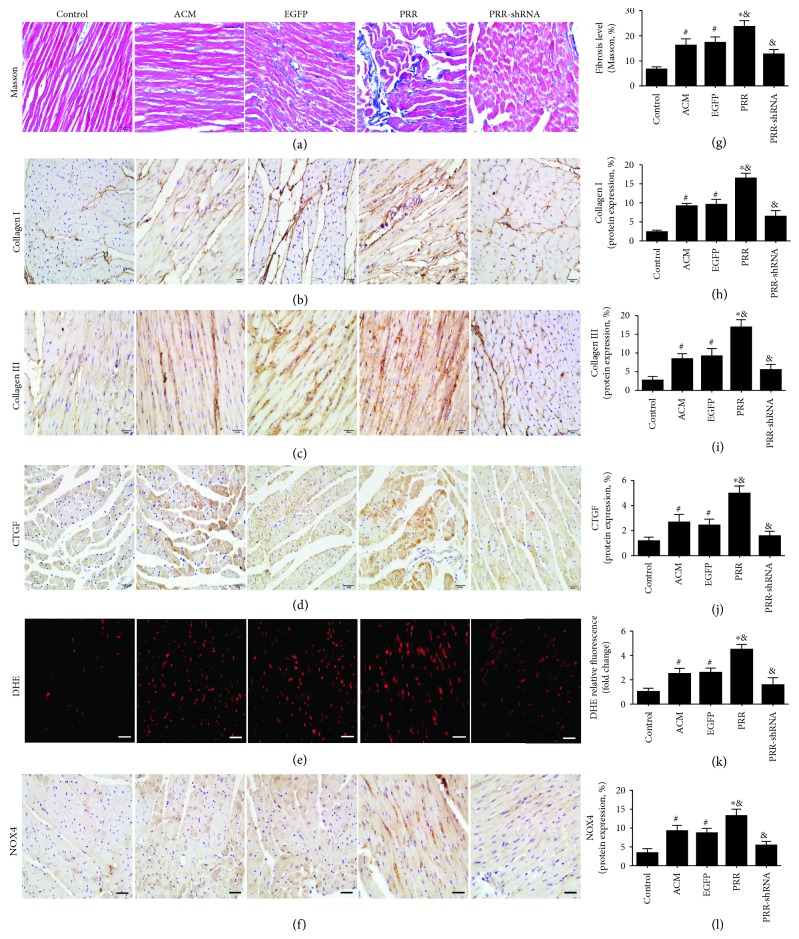
Levels of myocardial fibrosis and oxidative stress in all rats groups. (a)–(d) Representative Masson's trichrome staining and immunohistochemical staining of collagen I, collagen III, and connective tissue growth factor (CTGF) (scale bar = 20 *μ*m). (e) Representative dihydroethidium (DHE; 10 *μ*M) fluorescence (scale bar = 20 *μ*m). (f) Representative analysis of oxidative stress by immunohistochemical staining of NADPH oxidase 4 (NOX4). (g)–(l) Quantitative analysis of fibrosis level, collagen I, collagen III, connective tissue growth factor (CTGF) protein expression, DHE relative fluorescence, and NOX4 protein expression ^∗^ *P* < 0.01 and ^#^ *P* < 0.05 versus the control group. ^&^ *P* < 0.01 versus the ACM group.

Our results showed that alcohol consumption increased fibrillar collagen accumulation within the myocardium. Compared with the control group, the deposition of the extracellular matrix (ECM) was higher in the ACM and EGFP groups as indicated by Masson's trichrome staining (Figure 3(a)). Furthermore, PRR overexpression in the PRR group aggravated the deposition of ECM, compared with the ACM and EGFP groups (Figure 3(a)). Meanwhile, there was no statistical difference between the ACM group and the EGFP group. Immunohistochemical staining results revealed that PRR overexpression in the PRR group increased the protein expression of collagen I, collagen III, and connective tissue growth factor (CTGF), in contrast to the ACM and EGFP groups (Figures 3(b)–3(d)). The deposition of ECM and the expression of fibrotic factors in the PRR-shRNA group were significantly lower than those in the ACM group (Figures 3(a)–3(d)).

We also measured in vivo myocardial oxidative stress levels using DHE staining and observed an increased production of superoxide. Compared with the control group, alcohol consumption increased the production of superoxide and upregulated the expression of nicotinamide adenine dinucleotide phosphate oxidase 4 (NOX4) (Figures 3(e) and 3(f)). Furthermore, PRR overexpression in the PRR group increased superoxide production and NOX4 protein expression levels compared with the ACM and EGFP groups (Figures 3(e) and 3(f)). In addition to alleviating fibrosis, PRR silencing reduced the production of superoxide and the expression of NOX4 induced by alcohol consumption (Figures 3(e) and 3(f)).

### 3.4. ERK Kinase Inhibitor and Telmisartan Ameliorated Oxidative Stress Induced by PRR Overexpression In Vitro in Neonatal Cardiac Fibroblasts (CFs)

**Figure 4:**
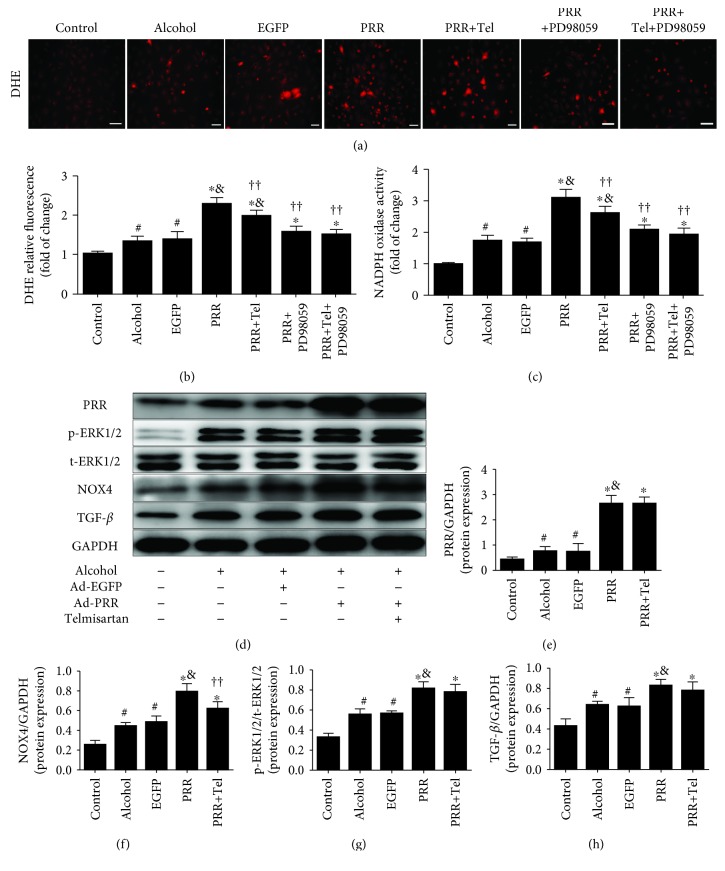
Oxidative stress; protein expression levels of PRR, NOX4, and TGF-*β*; and phosphorylation of ERK1/2 after PRR gene transfer in primary neonatal cardiac fibroblasts (CFs). (a) Representative dihydroethidium (DHE) fluorescence image of CFs (scale bar = 20 *μ*m). (b, c) Statistical results of oxidative stress by DHE relative fluorescence and NADPH oxidase activity. (d) Representative western blot analysis of ERK1/2 phosphorylation and PRR, NOX4, and TGF-*β* protein expression. (e)–(h) Quantification of protein expression levels of PRR/GAPDH, NOX4/GAPDH, p-ERK1/2/t-ERK1/2, and TGF-*β*/GAPDH. ^∗^ *P* < 0.01 and ^#^ *P* < 0.05 versus the control group, ^&^ *P* < 0.01 versus the EGFP group, and ^††^ *P* < 0.05 versus the PRR group. Tel: telmisartan.

PRR overexpression significantly exacerbated alcohol-induced oxidative stress of CFs. Specially, superoxide production and NADPH oxidase activity were further increased in the PRR group compared with the alcohol and EGFP groups (Figures 4(a)–4(c)). There was no statistical difference between the alcohol group and the EGFP group. Compared with the PRR group, telmisartan alleviated oxidative stress of CFs by decreasing the production of superoxide and NADPH oxidase activity (Figures 4(a)–4(c)). Although telmisartan had no effect on phosphorylation of ERK1/2, it reduced the protein expression of NOX4 induced by PRR overexpression (Figure 4(d)).

**Figure 5:**
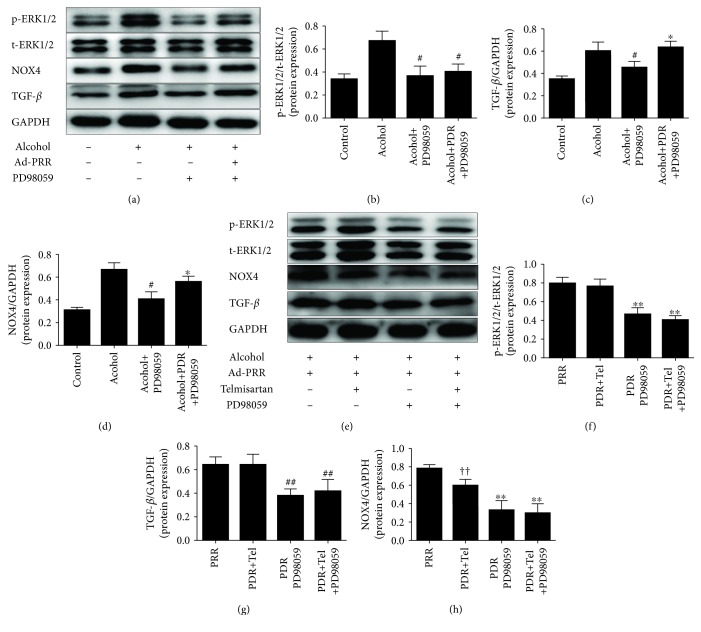
Effects of ERK kinase inhibitor (PD98059) and telmisartan on ERK1/2 phosphorylation and NOX4 and TGF-*β* protein expression in CFs. (a) and (e) Representative western blot analysis of ERK1/2 phosphorylation and NOX4 and TGF-*β* protein expression. (b)–(d) and (f)–(h) Quantification of protein expression level of p-ERK1/2/t-ERK1/2, TGF-*β*/GAPDH, and NOX4/GAPDH. ^#^ *P* < 0.05 versus the alcohol group, ^∗^ *P* < 0.05 versus the alcohol+PD98059 group, ^∗∗^ *P* < 0.01 and ^##^ *P* < 0.05 versus the PRR+Tel group, and ^††^ *P* < 0.05 versus the PRR group. Tel: telmisartan.

However, the ERK kinase inhibitor (PD98059) not only decreased the phosphorylation of ERK1/2 (Figure 5(e)) but also alleviated oxidative stress and NOX4 protein expression induced by PRR overexpression in CFs (Figures 4(a)–4(c) and 5(e)). According to our results, the effect of PD98059 was better than that of telmisartan in terms of reducing oxidative stress, and the combination of the two can produce effects that are more prominent.

### 3.5. ERK Kinase Inhibitor Reduced In Vitro ERK1/2 Phosphorylation and the Production of NOX4 and TGF-*β* with (without) PRR Overexpression in Neonatal Cardiac Fibroblasts (CFs)

Our results revealed that alcohol stimulation alone upregulated ERK1/2 phosphorylation and NOX4 and TGF-*β* protein expression in CFs. PRR overexpression further aggravated these effects (Figure 4(d)), while PD98059 reversed the phosphorylation of ERK and the expression of NOX4 and TGF-*β* (Figure 5(a)). There were no statistical differences in the expression of NOX4 and TGF-*β* between the alcohol+PRR+PD98059 and alcohol groups (Figure 5(a)). Correspondingly, we compared PD98059 and telmisartan in terms of ERK phosphorylation, NOX4, and TGF-*β* expression caused by PRR overexpression. According to our results, PD98059 is superior to telmisartan in decreasing ERK phosphorylation and NOX4 and TGF-*β* expression caused by PRR overexpression (Figure 5(e)).

### 3.6. Silencing of PRR Protein Expression Ameliorated In Vitro Oxidative Stress, Phosphorylation of ERK1/2, and Reduced NOX4 Protein Expression in Neonatal Cardiac Fibroblasts (CFs)

**Figure 6:**
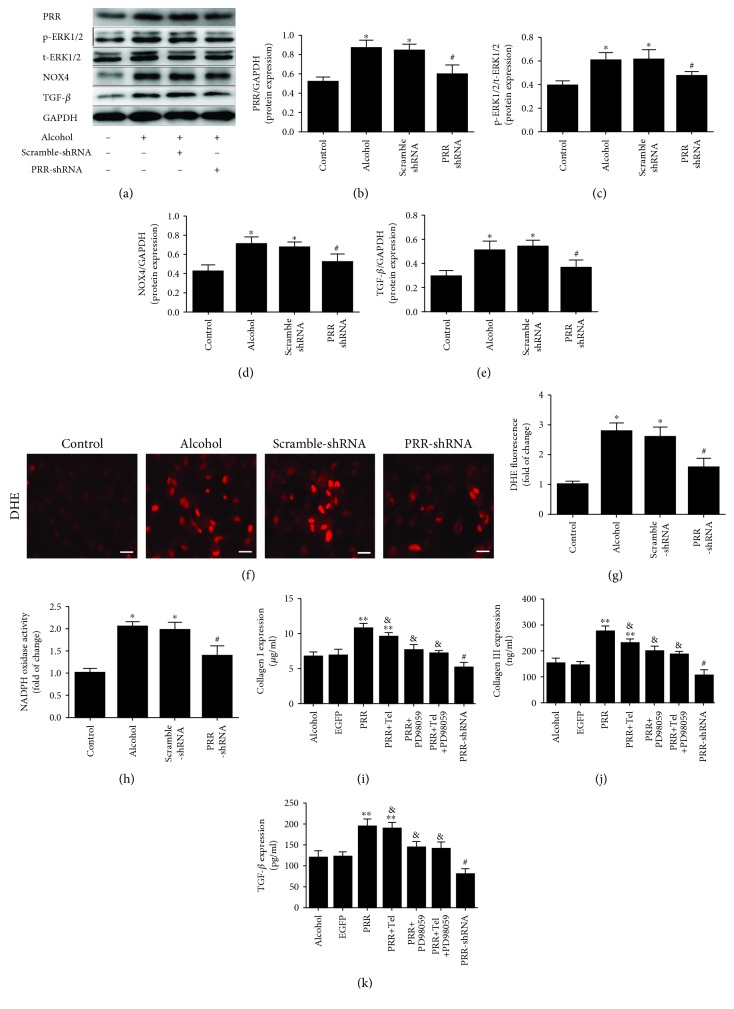
Oxidative stress, release of fibrosis factors after silencing PRR protein expression in primary neonatal cardiac fibroblasts (CFs). (a) Representative western blot analysis of PRR, ERK1/2 phosphorylation, and NOX4 and TGF-*β* protein expression in CFs. (b)–(e) Quantification of PRR/GAPDH, p-ERK1/2/t-ERK1/2, NOX4/GAPDH, and TGF-*β*/GAPDH protein expression levels. (f)–(h) Representative oxidative stress by DHE relative fluorescence and NADPH oxidase activity in CFs. (i)–(k) ELISA measurement of culture media levels of collagen I, collagen III, and TGF-*β* expression levels in all groups of CF. ^∗^ *P* < 0.01 versus the control group, ^#^ *P* < 0.05 versus the Scramble-shRNA (or EGFP) group, ^∗∗^ *P* < 0.01 versus the alcohol group, and ^&^ *P* < 0.05 versus the PRR group.

In the present study, we used PRR-shRNA technology to silence the expression of PRR. As our data revealed, CFs transfected with PRR-shRNA showed a lower expression of PRR protein in the PRR-shRNA group compared with the Scramble-shRNA group (Figure 6(a)). In addition, there was no significant difference between the alcohol group and the Scramble-shRNA group. Transfection with PRR-shRNA significantly decreased superoxide production and NADPH oxidase activity compared with the alcohol and Scramble-shRNA groups (Figures 6(f)–6(h)). Meanwhile, the elevated levels of ERK1/2 phosphorylation and NOX4 protein expression were inhibited in the PRR-shRNA group compared to the alcohol and Scramble-shRNA groups (Figure 6(a)).

### 3.7. Silencing of PRR Protein Expression Reduced the In Vitro Production of Profibrotic Factors

Alcohol stimulation increased TGF-*β* protein expression, but this effect could be prevented by silencing PRR protein expression (Figures 6(a) and 6(k)). There was no significant difference between the alcohol group and the Scramble-shRNA group. Similarly, silencing PRR protein expression decreased the protein expression of collagen I and collagen III (Figures 6(i) and 6(j)).

## 4. Discussion

In the present study, in vivo experiments revealed that overexpression of the (pro)renin receptor in rat alcoholic cardiomyopathy triggered a deposition of the extracellular matrix (ECM) and deteriorated cardiac function. These pathological changes were associated with phosphorylation of ERK1/2 and the upregulation of NOX4 protein expression. In addition, shRNA-mediated silencing of PRR reduced myocardial fibrosis. In vitro experimental results showed that the ERK kinase inhibitor (PD98059) was more effective than telmisartan in alleviating oxidative stress and reducing the production of profibrotic factors in neonatal cardiac fibroblasts (CFs). A previous study investigated the relationship between myocardial remodeling and PRR by in situ injection of a PRR gene into the anterior wall of the heart [23]. However, this study did not elaborate on the relationship between PRR and fibroblasts, which are a class of cells that are active during myocardial remodeling. Therefore, we conducted more detailed cellular gain-of-function and loss-of-function experiments to demonstrate that PRR is involved in the progression of myocardial fibrosis.

Previous studies showed that the renin-angiotensin system, particularly angiotensin II, plays an important role in various kinds of cardiovascular diseases [5, 24]. The RAS blockers, angiotensin II type 1 receptor blocker (ARB), and angiotensin-converting enzyme inhibitor (ACEI) have been widely used to reduce end-organ damage. However, these therapeutic strategies do not completely prevent pathological processes [25, 26]. The reasons for the controversial therapeutic strategy have been troubling for researchers. Therefore, new treatment regimens targeting the RAS should be proposed to achieve a more optimal clinical outcome. Angiotensin-converting enzyme 2 (ACE2) has been widely studied as an important modulator of the RAS. Our previous study revealed that ACE2 overexpression improved left ventricular remodeling and function in diabetic cardiomyopathy [22]. We also demonstrated that ACE2 overexpression significantly inhibited early atherosclerotic lesions by improving endothelial cell function and suppressing vascular smooth muscle cell proliferation and migration [27]. The main mechanism of ACE2 involves the cleavage of angiotensin II (Ang II) to Ang (1–7) and inhibition of ACE function.

The (pro)renin receptor is a multifunctional receptor consisting of 350 amino acids with a molecular weight of approximately 37-39 kD. By binding to (pro)renin and/or renin, PRR can fully activate (pro)renin and increase the catalytic activity of renin, which can further increase the production of Ang II and produce a series of biological effects. On the other hand, this binding also activates PRR, which further activates intracellular signals independent of Ang II. In fact, local tissue RAS has attracted increasing attention in end-organ damage compared to circulating RAS. PRR, a key functional component of local tissue RAS, was first cloned in 2002, and its pathophysiological role in disease processes has been extensively studied since. Shi et al. reported that PRR could activate NF-*κ*B and increase the expression of proinflammatory cytokines in microglial cells [28]. Additionally, there is strong evidence indicating that PRR is also expressed in kidney tissues [29, 30]. For example, conditional deletion of PRR in nephron progenitor cells resulted in podocyte abnormalities and proteinuria [31]. Meanwhile, PRR activation has been reported to increase the production of profibrotic markers and aggravate renal tubular injury [32]. In the hearts of diabetic rats, (pro)renin receptor mRNA and protein were significantly increased, promoting myocyte hypertrophy, interstitial fibrosis, and worsening cardiac function [33]. There is no doubt that PRR plays a key role in the local tissue RAS. Available evidence suggests that PRR executes its biological effects primarily through the angiotensin II- (Ang II-) independent pathway [24]. In a mouse model of ischemia-reperfusion injury, overexpression of PRR aggravated renal inflammation and fibrotic lesions by amplifying Wnt/*β*-catenin signaling rather than relying on Ang II [32]. In postmyocardial infarct hearts, PRR increased myocardial fibrosis and worsened cardiac function, and these effects did not depend on Ang II [34].

As our study revealed, overexpression of the PRR gene in alcoholic cardiomyopathy model rats induced severe myocardial fibrosis and dysfunction. Myocardial fibrosis is one of the major predisposing factors for the development of cardiac dysfunction. Irrespective of etiology, cardiac fibrosis increases the risk of cardiac morbidity and mortality in end-stage heart failure [35]. Our results showed that cardiac fibrosis caused by PRR overexpression was associated with increased gene expression of collagen I, collagen III, and CTGF. Our results also showed that PRR overexpression increased the production of alcohol-induced collagen and TGF-*β* in primary neonatal cardiac fibroblasts. Among several cell types, including mesangial cells, collecting duct cells and vascular smooth muscle cells [24], PRR activated the ERK1/2-MAPK family. Activation of ERK1/2 can increase cell proliferation and production of TGF-*β*, leading to the production of profibrotic factors such as collagen and PAI-1 [36, 37]. In our study, we found the similar profibrotic biological effects of PRR in CFs. Although telmisartan reduced the production of collagen I and collagen III induced by PRR overexpression in CFs, it had no significant effect on the production of TGF-*β* or the phosphorylation of ERK1/2-MAPK. These data indicated that the production of collagen induced by PRR overexpression was partly dependent upon the Ang II pathway, whereas ERK1/2-MAPK was directly phosphorylated by PRR independent of Ang II. Although previous studies have focused on the effects of the PRR-Ang II-dependent pathway, surging data has recently shown that the PRR-Ang II-independent pathway usually plays a more important role [38, 39].

Oxidative stress is also involved in the pathological process of ACM [5]. As a common source of oxidative stress-NADPH oxidase (NOX), there are seven NOX isoforms in mammals [40]. Among these isoforms, NOX1, NOX2, and NOX4 are abundantly expressed in hearts [41]. Acetaldehyde, formed by alcohol dehydrogenase, is the major biochemical cardiotoxic substance. Accumulated acetaldehyde induced by chronic alcohol intake upregulates the protein expression of NOX2 and its subunits, which increase mitochondrial-derived ROS in cardiomyocytes [42]. It is acceptable that overexpression of aldehyde dehydrogenase-2 reduces myocardial damage in alcoholic cardiomyopathy [43]. Different from cardiomyocytes, our results showed that PRR overexpression upregulated NOX4 protein expression, relative to NOX1 and NOX2, which mediates differentiation of cardiac fibroblasts into myofibroblasts [44]. NOX–derived reactive oxygen species take part in many kinds of cardiovascular diseases. It has been reported that NADPH oxidase-derived superoxide is present in repetitive pulmonary embolism which leads to pulmonary arterial hypertension [45]. Kuroda et al. reported that the NOX4-ROS pathway had been shown to aggravate profibrotic responses, remodeling processes, and worsening cardiac function in the failing heart [46]. Pan et al. found that knockdown of NOX4 or ROS scavengers attenuated intercellular ROS generation and myocardial fibrotic responses in cardiac fibroblast stimulated by Ang II [47]. Based on these studies, we speculate that ROS derived from NOX4 plays a key role in cardiac fibrosis and myocardial damage. Both PD98059 and telmisartan reduced NOX4 expression and the production of intracellular ROS, suggesting that oxidative stress may involve multiple pathways. There are several sources of ROS production [41], and in particular, Ang II can enhance the physical association between AT1 and NOX4 [48]. Similarly, PRR-shRNA could also reduce DHE fluorescence, NADPH oxidase activity, and NOX4 protein expression. In fact, there is growing evidence showing that NOX4 is constitutively active and its function does not depend on combining with other cytosolic subunits [49]. As our data showed, PRR overexpression may upregulate NOX4 expression primarily through the PRR-ERK1/2 pathway rather than a single Ang II pathway.

In conclusion, alcohol consumption can cause myocardial remodeling and eventually lead to maladaptive cardiac function [50]. We found that increased expression of PRR was accompanied by the development of ACM. The (pro)renin receptor is a pivotal functional member of the local tissue RAS and plays an important role in the development of ACM through ROS derived from the PRR-ERK1/2-NOX4 pathway. Our study reveals a potential therapeutic strategy for alleviating oxidative stress, reducing cardiac fibrosis, and improving cardiac function in alcoholic cardiomyopathy.

## Data Availability

The data used to support the findings of this study are available from the corresponding author upon request.

## Supplementary Materials

Supplementary Materials The PRR signals in ROS production and fibrosis. In cardiac fibroblasts, PRR enhances the expression of NOX4 via Ang II-AT1 and the PRR-ERK1/2-NOX4 pathway, activates the NADPH oxidase activity, and increases ROS production and fibrosis. Abbreviations: PRR: (pro)renin receptor; AT1: angiotensin II type 1 receptor; NOX4: nicotinamide adenine dinucleotide phosphate oxidase 4: ERK-MAPK: extracellular signal-regulated kinase-mitogen-activated protein kinase; ROS: reactive oxygen species.
