# Supplementary figures and images for: Effects of the (Pro)renin Receptor on Cardiac Remodeling and Function in a Rat Alcoholic Cardiomyopathy Model via the PRR-ERK1/2-NOX4 Pathway
Source: Oxid Med Cell Longev. 2019 Mar 13;2019:4546975. doi: 10.1155/2019/4546975 (PMC6462324; doi:10.1155/2019/4546975)

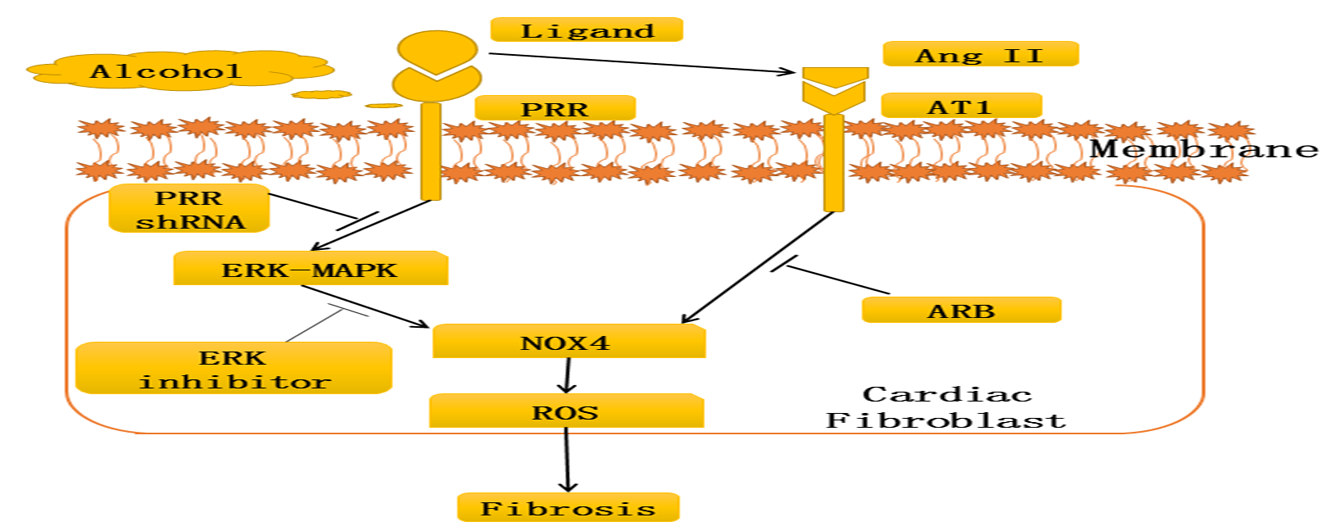

Supplement: Supplementary Materials — The PRR signals in ROS production and fibrosis. In cardiac fibroblasts, PRR enhances the expression of NOX4 via Ang II-AT1 and the PRR-ERK1/2-NOX4 pathway, activates the NADPH oxidase activity, and increases ROS production and fibrosis. Abbreviations: PRR: (pro)renin receptor; AT1: angiotensin II type 1 receptor; NOX4: nicotinamide adenine dinucleotide phosphate oxidase 4: ERK-MAPK: extracellular signal-regulated kinase-mitogen-activated protein kinase; ROS: reactive oxygen species. [file 4546975.f1.tif]
